# Predictors and Outcomes Associated with Bariatric Robotic Delivery: An MBSAQIP Analysis of 318,151 Patients

**DOI:** 10.3390/jcm13144196

**Published:** 2024-07-18

**Authors:** Khadija Nasser, Sukhdeep Jatana, Noah J. Switzer, Shahzeer Karmali, Daniel W. Birch, Valentin Mocanu

**Affiliations:** 1 Department of Surgery, University of Alberta, Dvorkin Lounge Mailroom 2G2 Walter C. Mackenzie Health Sciences Centre, 8440-112 ST NW, Edmonton, AB T6G 2B7, Canada; knasser@ualberta.ca (K.N.); vmocanu@ualberta.ca (V.M.); 2 Centre for Advancement of Surgical Education and Simulation (CASES), Royal Alexandra Hospital, Edmonton, AB T5H 3V9, Canada

**Keywords:** sleeve gastrectomy, Roux-en-Y gastric bypass, robotic surgery, minimally invasive surgery, outcomes, patient selection

## Abstract

**Background**: The adoption of robotic bariatric surgery has increased dramatically over the last decade. While outcomes comparing bariatric and laparoscopic approaches are debated, little is known about patient factors responsible for the growing delivery of robotic surgery. A better understanding of these factors will help guide the planning of bariatric delivery and resource allocation. **Methods**: Data were extracted from the MBSAQIP registry from 2020 to 2021. The patient population was organized into primary robot-assisted sleeve gastrectomy or Roux-en-Y gastric bypass (RYGB) versus those who underwent laparoscopic procedures. Bivariate analysis and multivariable logistic regression modeling were conducted to characterize cohort differences and identify independent patient predictors of robotic selection. **Results**: Of 318,151, 65,951 (20.7%) underwent robot-assisted surgery. Patients undergoing robotic procedures were older (43.4 ± 11.8 vs. 43.1 ± 11.8; *p* < 0.001) and had higher body mass index (BMI; 45.4 ± 7.9 vs. 45.0 ± 7.6; *p* < 0.001). Robotic cases had higher rates of medical comorbidities, including sleep apnea, hyperlipidemia, gastroesophageal reflux disease (GERD), and diabetes mellitus. Robotic cases were more likely to undergo RYGB (27.4% vs. 26.4%; *p* < 0.001). Robotic patients had higher rates of numerous complications, including bleed, reoperation, and reintervention, resulting in higher serious complication rates on multivariate analysis. Independent predictors of robotic selection included increased BMI (aOR 1.02), female sex (aOR 1.04), GERD (aOR 1.12), metabolic dysfunction, RYGB (aOR 1.08), black racial status (aOR 1.11), and lower albumin (aOR 0.84). **Conclusions**: After adjusting for comorbidities, patients with greater metabolic comorbidities, black racial status, and those undergoing RYGB were more likely to receive robotic surgery. A more comprehensive understanding of patient factors fueling the adoption of robotic delivery, as well as those expected to benefit most, is needed to better guide healthcare resources as the landscape of bariatric surgery continues to evolve.

## 1. Introduction

Bariatric surgery is the single most effective sustained long-term treatment for obesity and its associated metabolic comorbidities [1]. Rates of bariatric surgery have been steadily increasing over the last decade as the obesity epidemic continues to impose a tremendous burden on both local and global healthcare resources. Advancements in bariatric delivery, including the increased adoption of robotic surgery, have been recently adopted with the hopes of improving outcomes and streamlining surgical delivery [2]. Yet, while robotic bariatric surgery has been examined in multiple settings, including primary and revisional procedures, results have met with conflicting findings of benefit, with some studies suggesting possible harm [3,4,5]. A better understanding of these factors will help to better guide the planning of bariatric delivery and resource allocation. 

A recent study assessed complications associated with bariatric surgery and suggested factors that may alter decision-making; however, it did not comment on current factors associated with the selection of bariatric surgery [6]. Suggested benefits of robotic surgery include improved ergonomics and technique, increased degrees of freedom of movement, and avoiding the usage of bariatric-specific laparoscopic instruments. Furthermore, the recent literature has shown decreased complication rates with robotic platforms, such as leaks, hemorrhage, and stricture [4]. However, concerns about robotic surgery include the cost and increased duration of cases [7]. Further, there is a learning curve associated with robotic cases [8]. In the context of the high standards set by the Metabolic and Bariatric Surgery Accreditation and Quality Improvement Program (MBSAQIP) [9], this can be a difficult skill set and resource to uptake and implement successfully. Understanding which patients are being selected may allow for the identification of trends in practice and potential opportunities for optimizing patient selection.

Using the multicentre prospective Metabolic and Bariatric Surgery Accreditation and Quality Improvement Program (MBSAQIP) database, we aim to compare populations undergoing robotic versus laparoscopic surgery and assess for factors associated with selection for primary bariatric surgery delivery. The second aim is to provide an up-to-date analysis of comorbidity-adjusted complication rates between the two cohorts with respect to surgical modality.

## 2. Materials and Methods

### 2.1. Study Design and Data Source

A retrospective cohort study was conducted to determine predictive factors for patient selection to undergo robotic bariatric surgery versus a conventional laparoscopic approach. Our primary outcome was the identification of independent patient factors associated with undergoing robotic surgery. Secondary outcomes include a comparison of the rate and types of postoperative complications between the two groups.

Data were obtained from the Metabolic and Bariatric Surgery Accreditation and Quality Improvement Program (MBSAQIP) database. All patients who underwent sleeve gastrectomy (SG) or Roux-en-Y gastric bypass (RYGB) between 2020 and 2021 were included. The MBSAQIP database captures bariatric procedures performed in over 885 accredited bariatric centers across North America. Data were collected by trained professionals from and available to MBSAQIP-accredited centers and were subject to frequent reviews to maintain accuracy, with a target of data integrity audit disagreement rate of ≤5%. Detailed information about reporting of outcomes and collection of information is available in the Participant Use Data File [10]. Ethics approval was not sought for this study, given the nature of the database. Data were collected anonymously and stored in a secure database for patients of MBSAQIP centers.

### 2.2. Study Population and Variable Definitions

The patient population was organized into two cohorts: (1) those who underwent a primary robot bariatric procedure and (2) those who underwent a conventional laparoscopic procedure. Only RYGB and SG were evaluated, as these represent the vast majority of primary bariatric procedures performed. Patients undergoing other primary procedures and open procedures were excluded, along with revisional cases, as they represent different surgical challenges and are a different patient cohort.

Demographic and patient factors extracted included age, sex, body mass index (BMI), functional status (independent, partially dependent, and dependent prior to surgery), the American Society of Anesthesiologists (ASA) category, and smoking status. Comorbidities investigated included diabetes (non-diabetic/diet-controlled, non-insulin-dependent, and insulin-dependent), hypertension, gastroesophageal reflux disease (GERD), chronic obstructive pulmonary disease (COPD), hyperlipidemia, obstructive sleep apnea (OSA), venous stasis, prior venous thromboembolism (VTE), prior myocardial infarction (MI), and renal insufficiency. Additional comorbid factors included dialysis dependence, chronic steroid use, anticoagulation status, prior cardiac surgery, and prior percutaneous coronary intervention (PCI). Surgical factors assessed included year of operation and surgical approach. 

Outcomes investigated included postoperative complications such as leak, bleeding, cardiac complications, pneumonia, acute kidney injury (AKI), deep and superficial surgical site infections (SSI), wound disruption, sepsis, unplanned intubation, stroke, VTE, pulmonary embolism, and a composite variable of overall serious complications. The definition of our serious complications composite outcome is reoperation for, readmission for, reintervention for, or death due to an anastomotic leak or staple line leak or drain present 30 days postoperatively. Additionally, 30-day reoperation, intervention, readmission, and mortality rates were examined.

### 2.3. Statistical Analysis 

Baseline characteristics were described in a categorical and continuous fashion as appropriate. Analysis of categorical variables was performed using Chi-square tests, while the Kruskal–Wallis rank test was used for continuous variables. A multivariable logistic regression model was created to identify independent factors associated with the selected outcome after adjusting for comorbidities. The model used hypothesis-driven methodology to identify patient factors, as ascertained by proven factors associated with the selected outcome in the published literature [11,12,13], and also included variables with *p* < 0.10 on bivariate analysis for the main effects model. Multivariable models were used to identify factors associated with patients undergoing robotic surgery and for 30-day serious complications. Missing data would exclude the case from the model. To assess model fit, the Brier Score and area under the Receiver Operating Curves were calculated. Statistical analysis was performed using Stata 15 (STATACorp LP, College Station, TX, USA). 

## 3. Results

### 3.1. Study Population and Comparison of Baseline Characteristics

**Figure 1:**
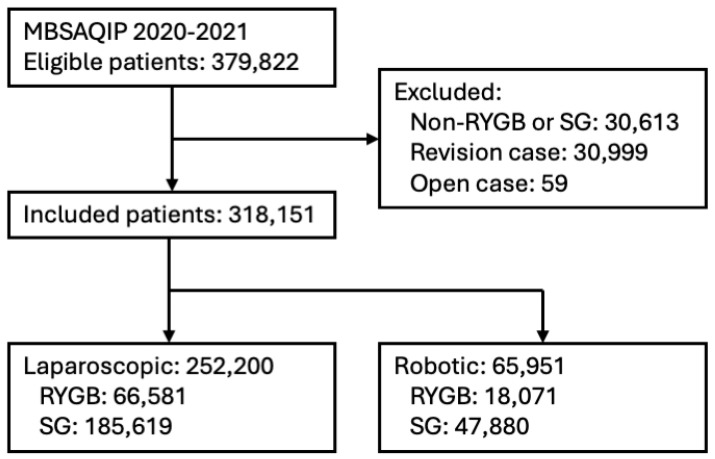
Patient flow diagram. Abbreviations: MBSAQIP, Metabolic and Bariatric Surgery Accreditation and Quality Improvement Program; RYGB, roux-en-Y gastric bypass; SG, sleeve gastrectomy.

Of 318,151 included patients, 65,951 (20.7%) underwent a robot-assisted primary bariatric procedure (Figure 1, Table 1), with the remaining undergoing a laparoscopic procedure. Patients undergoing robotic procedures were older (43.4 ± 11.8 years vs. 43.1 ± 11.8 years; *p* < 0.001) and had a higher BMI (45.4 ± 7.9 kg/m^2^ vs. 45.0 ± 7.6 kg/m^2^; *p* < 0.001). There were no differences in robotic delivery with respect to functional status or female sex (82.1% vs. 81.8%, *p* = 0.065). Generally, robotic cases had higher rates of metabolic comorbidities, including hypertension (45.9% vs. 43.2%, *p* < 0.001), COPD (1.4% vs. 1.1%, *p* < 0.001), sleep apnea (38.0% vs. 36.8%, *p* < 0.001), hyperlipidemia (23.5% vs. 21.7%, *p* < 0.001), GERD (32.4% vs. 29.8%, *p* < 0.001), and medication-dependent diabetes (23.7% vs. 22.6%; *p* < 0.001). Patients undergoing a robotic procedure also had higher presurgical ASA classifications (4.2% vs. 3.6% ASA category 4–5; *p* < 0.001), chronic steroid use (2.5% vs. 2.1%; *p* < 0.001), and increased rates of prior VTE (2.7% vs. 2.4%; *p* < 0.001).

Surgical procedural selection and operative length also differed between the two cohorts. The majority of patients underwent SG (73.3%), with robotic cases more likely to undergo RYGB (27.4% vs. 26.4%; *p* < 0.001). Operative length was significantly greater in robotic cases vs. laparoscopic procedures (104.9 ± 53.1 min vs. 79.1 ± 46.8 min; *p* < 0.001). Furthermore, the number of robotic cases increased from 2020 to 2021 from 24,705 procedures to 41,246 procedures (17.5% to 23.3% of the included cohort; *p* < 0.001). 

### 3.2. Comparison of Postoperative Complications in Robotic vs. Laparoscopic Cohorts

To determine the impact of robotic cases on the development of postoperative complications, we compared complication rates between laparoscopic and robotic cases (Table 2). In comparison to the laparoscopic cohort, robotic cases had higher rates of overall serious complication (3.2% vs. 2.8%, *p* < 0.001) and deep organ space infection (0.4% vs. 0.3%, *p* = 0.010). Rates of superficial surgical site infection (0.3% vs. 0.3%; *p* = 0.736) and deep surgical site infections (0.1% vs. 0.0%; *p* = 0.615) between robotic and laparoscopic cases did not differ. Despite no difference in leak rates (0.3 vs. 0.2%; *p* = 0.1), anastomotic/staple line bleed rates were higher in the robotic cohort (1.0 vs. 1.0%; *p* = 0.001), as were 30-day reoperation (1.0 vs. 0.9%; *p* = 0.003), reintervention (0.8% vs. 0.7%; *p* = 0.016), and readmission rates (3.3 vs. 2.8%; *p* < 0.001). There were no significant differences in rates of cardiac complications (0.1% vs. 0.1%; *p* = 0.3), cerebrovascular accidents (0.0% vs. 0.0%; *p* = 0.4), or acute kidney injury (0.1% vs. 0.1%; *p* = 0.8). Further, no clinically important differences were observed between rates of pneumonia, sepsis, or thromboembolic disease. 

### 3.3. Multivariable Model Predicting 30-Day Serious Complications

Numerous variables in the multivariate logistic regression model were predictive of 30-day serious complications (Table 3). Demographic factors, including older age (aOR 1.06, 95% CI 1.05–1.09; *p* < 0.001) and black race (aOR 1.30, 95% CI 1.20–1.33; *p* < 0.001), were associated with increased odds of serious complications. Notably, undergoing robotic surgery was not independently associated with an increased risk of 30-day serious complications (aOR 1.04, 95% CI 0.99–1.90; *p* = 0.099). Co-variates that most contributed to the development of 30-day serious complications after adjustment were fully dependent functional status (aOR 2.24, 95% CI 1.01–5.78; *p* = 0.047), renal insufficiency (aOR 2.00, 95% CI 1.96–2.78; *p* < 0.001), and undergoing RYGB (vs. SG; aOR 2.06, 95% CI 1.96–2.16; *p* < 0.001). The Brier score was 0.028, and the ROC area was 0.66, indicating a good model fit.

### 3.4. Multivariable Model Assessing Robotic Surgery Selection 

A multivariable logistic regression model was also performed to assess for factors associated with undergoing robotic surgery (Table 4). Numerous demographic factors were associated with robotic surgery, including higher likelihood in females (aOR 1.04, 95% CI 1.02–1.07; *p* = 0.001) and black racial status (aOR 1.11, 95% CI 1.09–1.14; *p* < 0.001). Patients with increasing metabolic burden are more likely to undergo robotic surgery, such as higher BMI (aOR 1.02, 95% CI 1.01–1.02; *p* < 0.001) and lower albumin (aOR 0.85, 95% CI 0.82–0.86; *p* < 0.001). Other metabolic comorbidities predictive of robotic selection include hyperlipidemia (aOR 1.07, 95% CI 1.04–1.10; *p* < 0.001), GERD (aOR 1.12, 95% CI 1.09–1.14; *p* < 0.001), hypertension (aOR 1.07, 95% CI 1.05–1.09; *p* < 0.001), non-insulin-dependent diabetes mellitus (aOR 1.03, 95% CI 1.00–1.06; *p* = 0.024), COPD (aOR 1.20, 95% CI 1.11–1.31; *p* < 0.001), dialysis dependence (aOR 1.22, 95% CI 1.03–1.43, *p* = 0.020), and chronic steroid (aOR 1.12, 95% CI 1.05–1.20; *p* < 0.001). In terms of procedure, those undergoing RYGB were more likely to undergo a robotic procedure compared to SG (aOR 1.08, 95% CI 1.06–1.10; *p* < 0.001). The Brier Score and area under ROC for this model were 0.061 and 0.060, suggesting an acceptable model fit.

## 4. Discussion

The adoption of robotic surgery has been increasing globally in all aspects of general surgery, with bariatric surgery following suit [14]. Despite studies assessing how the selection of patients can affect postoperative outcomes [6], there is still limited information known about patient selection for robotic bariatric procedures. After adjusting for comorbidities, patients with a greater burden of metabolic comorbidities and frailty and those undergoing RYGB were more likely to undergo robotic bariatric surgery. Despite these results, there were still no strongly contributing single patient factors predictive for robotic selection, suggesting selection may be independent of patient factors and based instead on system factors such as robotic availability at individual centers or patient preference. Lastly, with the use of the newly updated MBSAQIP data, it was shown that robotic bariatric surgery is not associated with adverse adjusted outcomes as was suggested in the previous literature [15].

It is unclear whether these higher-risk patients (e.g., increased comorbidities and frailty) would benefit from robotic surgery or how they would compare to laparoscopy-based procedures. Benefits of robotic-assisted cases include improved ergonomics through allowance of increased degrees of freedom [16], especially in cases requiring multiple anastomoses (e.g., RYGB). Furthermore, some bariatric cases may be accompanied by concurrent paraesophageal hernia repairs [17]. Given the technical difficulty of such cases, surgical providers may have chosen to preferentially use robotic approaches in such patients, which may explain the association of GERD with robotic selection. Despite these results, there were no strong single patient factors that were predictive for robotic selection. Further, factors associated with robotic surgery in our analysis, such as racial status, do not seem to be explained by the prior literature. This suggests that surgical decisions to proceed with a robotic approach may be relatively less dependent on underlying patient factors and instead be driven by system-level factors such as robotic availability and adoption of training across North American centers. This is consistent with the recent literature showing an immediate increase in robotic procedures juxtaposed with a decline of traditional laparoscopic approach across all disciplines of surgery in hospitals with newly launched robotic programs with often no clinical benefit of robotic platforms [14].

The robotic approach for bariatric procedures remains controversial due to the high cost versus lack of clear superiority with regard to clinical outcome benchmarks [18,19]. Results from this study show a 25 min time increase in operation compared to a laparoscopic approach. Increased operating time, along with the higher cost of robotic instruments [18,20], can be seen as barriers in a discipline where operating times are short–mid-length, and outcomes are already well-optimized [21]. A previous study showed that ASA class II and III patients perform similarly in terms of complication rate to patients of ASA class IV [21]. Practically, surgeon training in robotics programs and early uptake in residency and fellowship training programs may also be another obstacle to widespread implementation. 

Despite this, studies have shown consistent yearly increases in the adoption of a robotic approach in bariatric cases, also reflected in our data (Table 1), suggesting ongoing high surgical interest in performing robotic cases [2,14,22,23]. In a recent study by Bauerle et al., they demonstrated a steady increase in all types of bariatric procedures from 2015 to 2020, with the greatest annual increase in revisional bariatric surgery (RBS) followed by SG [2]. This is consistent with another study by Tartarian et al. demonstrating a 1.96-fold increase in robotic case load from 2015 to 2018 [23]. It has also been suggested that the interest and benefit in a robotic approach may be greater in those procedures with more technical challenges, such as anatomically challenging spaces or difficult anastomoses–factors that are difficult to account for in retrospective cohort studies [24]. Additionally, ergonomic factors are also thought to be responsible for the rising adoption of robotic techniques, given the greater physical toll on the surgical team with laparoscopic approaches. These purported benefits are thought to be drivers leading to an increased uptake in RBS [24,25].

Despite this increase in the uptake of robotic cases, there still remains debate regarding clinical outcome superiority, with the literature suggesting that robotic procedures may be associated with worse outcomes [22]. However, with the analysis of the newly updated MBSAQIP data, our study demonstrated that despite differences in various complication rates on bivariate analysis, robotic surgery did not appear predictive of 30-day serious complications on multivariate analysis after accounting for differences in baseline characteristics and other influences. This is consistent with more recent studies showing no significant difference in major and minor complications between robotic and laparoscopic approaches [19,26] and instead added benefit in complex bariatric procedures such as RBS [5]. Drawbacks of robotics not discussed in this study include technical failures such as device malfunctions, with rates varying between 0.4 and 8.0% [27]. Though this number is expected to improve with increased comfort, accessibility, and technology, its impact on centers first adapting robotics may be more substantial. 

The future of robotic bariatric surgery will likely change, with increased accessibility, expertise, and the multitude of acknowledged technical benefits of a robot platform. Similar proposed barriers were seen with robotic hepatobiliary [28] and colorectal [29] surgery, fields which are both showing increased utilization of robotics. Additionally, simulation programs in robotics can allow for more critical skills assessment and improved feedback during training exercises [29]. Our study noted that patients undergoing RYGB were more likely to be chosen for robotic-assisted procedures, likely due to the technical difficulty compared to SG. Thus, as other bariatric procedures begin to become more prominent, such as single anastomosis duodeno-ileal bypass with SG (SADI-S) [30] and biliopancreatic diversion with duodenal switch (BPD-DS) [31], and an increasing number of patients with a history of bariatric surgery may require revisional surgery, the uptake of robotics may simulate that in other abdominal surgery disciplines.

A limitation of this study is its retrospective cohort methodology. Retrospective studies may be impacted by unidentified confounders. As well, the database nature of the study is subject to the risk of selection bias, and the impact of center-specific practices or outcomes may not be captured. However, data are collected prospectively and reviewed frequently, and MBSAQIP remains the largest database for bariatric surgery patients in North America. In addition, as data were collected predominantly from North American populations, where rates of obesity are higher, the results may be less generalizable to Eastern populations. Further, our study only examined 30-day outcome measures following bariatric procedures and does not consider possible long-term outcomes or complications of robotic surgery compared to laparoscopic procedures. There is also a lack of variables in the database to assess center-specific outcomes, as there may be centers practicing exclusively one technique while others may practice both laparoscopy and robotic procedures. With the novelty of robotic bariatric surgery, there are also limited data available regarding surgeon training and exposure in robotic cases, which may affect results.

Nonetheless, this large database-based cohort study is the first to assess factors predictive of undergoing robot-assisted bariatric surgery. The results of this study, both regarding factors predictive of robotic bariatric surgery selection and patient complication rates, can help guide clinical decision-making and generate additional hypotheses for future studies. By demonstrating no significant difference in serious complications, clinicians may feel more comfortable selecting patients for robotic surgery based on technical and operative factors rather than patient factors. Furthermore, certain cohorts were found to be more likely to undergo robotic surgery, which is traditionally considered higher risk (such as those with increased comorbidities). These cohorts would benefit from further research assessing the benefit of undergoing robotic surgery (e.g., the wound infection rate in immunocompromised undergoing robotic versus laparoscopic cases).

Future studies can assess postoperative outcomes and complications in patients deemed high risk when undergoing robotic vs. laparoscopic surgery to help with selecting patients who would benefit most from robot-assisted surgery. Further, studies focusing on short- and long-term complications with emphasis on identifying patient cohorts who are most likely to benefit from robotic surgery would help with better delineating the ideal patients for robotic procedural selection

## 5. Conclusions

This study shows increasing utilization of robot-assisted primary bariatric procedures with patients of black racial status, increased comorbidities, and those undergoing RYGB more likely to undergo robotic-assisted surgery. Further, robot-assisted procedures were associated with an increased risk of complications on univariate but not multivariate analysis.

## Figures and Tables

**Table 1:** Baseline patient characteristics of patients undergoing robotic surgery versus laparoscopic surgery. Significant values are italicized. Data represented as n (%) unless otherwise stated.

	Laparoscopic (n = 252,200)	Robotic (n = 65,951)	*p*-Value
Age, years (mean ± SD)	43.1 ± 11.8	43.4 ± 11.8	*<0.001*
<18	571 (0.2)	110 (0.2)	
18–30	31,262 (12.4)	7728 (11.7)	
30–40	71,483 (28.3)	18,728 (28.4)	
40–50	72,787 (28.9)	18,976 (28.8)	
50–60	52,115 (20.7)	13,817 (21.0)	
>60	23,982 (9.5)	6592 (10.0)	
Female	206,300 (81.8)	54,153 (82.1)	0.065
BMI, kg/m^2^ (mean ± SD)	45.0 ± 7.6	45.4 ± 7.9	*<0.001*
<35	9374 (3.72)	2210 (3.35)	
35–40	58,058 (23.0)	14,846 (22.5)	
40–50	78,822 (31.3)	20,205 (30.6)	
50–60	52,018 (20.6)	13,495 (20.5)	
60–70	42,646 (16.9)	11,719 (17.8)	
>70	11,280(4.5)	3473 (5.3)	
Functional status preoperatively			*0.034*
Fully independent	250,727 (99.5)	65,539 (99.4)	
Partially dependent	1180 (0.5)	348 (0.5)	
Fully dependent	43 (0.0)	18 (0.0)	
ASA class			*<0.001*
1–2	48,546 (19.3)	11,835 (17.0)	
3	194,181 (77.2)	51,270 (77.8)	
4–5	8938 (3.6)	2797 (4.2)	
Preoperative albumin (g/dL)	4.2 ± 0.4	4.1 ± 0.4	*<0.001*
Smoker	17,263 (6.85)	4323 (6.6)	*0.008*
Diabetes mellitus			*<0.001*
Non-diabetic or diet-controlled	195,267 (77.4)	50,303 (76.3)	
Insulin-independent	41,162 (16.3)	11,320 (17.2)	
Insulin-dependent	15,771 (6.3)	4328 (6.6)	
Hypertension	108,973 (43.2)	30,258 (45.9)	*<0.001*
GERD	73,564 (29.2)	21,335 (32.4)	*<0.001*
COPD	2767 (1.1)	924 (1.4)	*<0.001*
Hyperlipidemia	54,622 (21.7)	15,506 (23.5)	*<0.001*
Chronic steroids	5170 (2.05)	1631 (2.5)	*<0.001*

Abbreviations: ASA, American Society of Anesthesiologists; BMI, body mass index; COPD, chronic obstructive pulmonary disorder; GERD, gastroesophageal reflux disease.

**Table 2:** Complication rate by 30 days, stratified by method of surgery, robotic versus laparoscopic. Significant values are italicized. Data represented as n (%) unless otherwise stated.

	Laparoscopic (252,200)	Robotic (n = 65,951)	*p*-Value
Leak	568 (0.2)	171 (0.3)	0.106
Bleed	2214 (1.0)	667 (1.0)	*0.001*
Reoperation	2304 (0.9)	685 (1.0)	*0.003*
Reintervention	1764 (0.7)	520 (0.8)	*0.016*
Readmission	7016 (2.8)	2198 (3.3)	*<0.001*
Any cardiac complication	265 (0.1)	80 (0.1)	0.260
Pneumonia	447 (0.2)	159 (0.2)	*0.001*
Deep surgical site infection	742 (0.3)	235 (0.4)	*0.010*
Sepsis	214 (0.1)	71 (0.1)	0.081
Surgical site infection			
Superficial	816 (0.3)	226 (0.3)	0.736
Deep	107 (0.0)	31 (0.1)	0.615
Organ space	742 (0.3%)	235 (0.4)	*0.010*
Wound disruption	122 (0.0)	33 (0.1)	0.745
Venous thromboembolism	913 (0.4)	277 (0.4)	*0.030*
Pulmonary embolism	273 (0.1)	91 (0.1)	*0.040*
Cerebrovascular accident	31 (0.0)	11 (0.0)	0.383
Acute kidney injury	127 (0.1)	35 (0.1)	0.783
Serious complication	6965 (2.8)	2114 (3.2)	*<0.001*

**Table 3:** Multivariable logistic regression for predicting serious complications. Significant *p*-values are italicized.

Predictors of Serious Complications *	Adjusted Odds Ratio	95% Confidence Interval	*p*-Value
Older age (per 10 years)	1.06	1.05–1.09	*<0.001*
Higher BMI (per 5 kg/m^2^)	0.99	0.98–1.01	0.870
Race (vs. white)			
Black	1.30	1.20–1.33	*<0.001*
Other	0.96	0.90–1.02	0.208
Functional status (vs. fully independent)			
Partially dependent	1.83	1.50–2.24	*<0.001*
Fully dependent	2.42	1.01–5.78	*0.047*
Smoker	1.25	1.15–1.35	*<0.001*
Hyperlipidemia	1.06	1.00–1.12	*0.030*
Diabetes (vs. non-diabetic or diet-controlled)			
Non-insulin-dependent	0.97	0.92–1.03	0.388
Insulin-dependent	1.13	1.04–1.22	*0.002*
Hypertension	1.12	1.13–1.25	*<0.001*
COPD	1.47	1.27–1.69	*<0.001*
Renal Insufficiency	2.00	1.96–2.78	*<0.001*
History of VTE	1.87	1.70–2.06	*<0.001*
History of myocardial infarction	1.68	1.45–1.95	*<0.001*
RYGB (vs. SG)	2.06	1.96–2.16	*<0.001*
Operation length	1.00	1.00–1.00	*<0.001*
Robotic surgery (vs. laparoscopic)	1.04	0.99–1.90	0.099

Abbreviations: BMI, body mass index; COPD, chronic obstructive pulmonary disease; RYGB, Roux-en-Y gastric bypass; SG, sleeve gastrectomy; VTE, venous thromboembolism. * Definition of serious complication by 30 days: reoperation for, readmission for, reintervention for, or death due to anastomotic leak or staple line leak or drain present 30 days postoperatively.

**Table 4:** Multivariable logistic regression for factors predicting patients undergoing robotic surgery. Significant *p*-values are italicized.

Predictors of Robotic Surgery	Adjusted Odds Ratio	95% Confidence Interval	*p*-Value
Female	1.04	1.02–1.07	*0.001*
Higher BMI (per 5 kg/m^2^)	1.02	1.01–1.02	*<0.001*
Race (vs. white)			
Black	1.11	1.09–1.14	*<0.001*
Other	0.80	0.78–0.82	*<0.001*
Preoperative albumin	0.84	0.82–0.86	*<0.001*
GERD	1.12	1.09–1.14	*<0.001*
Hyperlipidemia	1.07	1.04–1.10	*<0.001*
Hypertension	1.07	1.05–1.09	*<0.001*
Diabetes (vs. non-diabetic or diet-controlled)			
Non-insulin-dependent	1.03	1.00–1.06	*0.024*
Insulin-dependent	0.97	0.93–1.02	0.217
COPD	1.20	1.11–1.31	*<0.001*
Dialysis dependence	1.22	1.03–1.43	*0.020*
Chronic steroids	1.12	1.06–1.20	*<0.001*
RYGB (vs. SG)	1.08	1.06–1.10	*<0.001*

Abbreviations: BMI, body mass index; COPD, chronic obstructive pulmonary disease; RYGB, Roux-en-Y gastric bypass; SG, sleeve gastrectomy; VTE, venous thromboembolism.

## Data Availability

The MBSAQIP database is accessible to MBSAQIP-designated centers.
